# Phytomicrobiome Coordination Signals Hold Potential for Climate Change-Resilient Agriculture

**DOI:** 10.3389/fpls.2020.00634

**Published:** 2020-05-25

**Authors:** Dongmei Lyu, Rachel Backer, Sowmyalakshmi Subramanian, Donald L. Smith

**Affiliations:** Department of Plant Science, Faculty of Agricultural and Environmental Sciences, McGill University, Montreal, QC, Canada

**Keywords:** phytomicrobiom, stress resiliance, biostimulants, crop, signal compounds

## Abstract

A plant growing under natural conditions is always associated with a substantial, diverse, and well-orchestrated community of microbes—the phytomicrobiome. The phytomicrobiome genome is larger and more fluid than that of the plant. The microbes of the phytomicrobiome assist the plant in nutrient uptake, pathogen control, stress management, and overall growth and development. At least some of this is facilitated by the production of signal compounds, both plant-to-microbe and microbe back to the plant. This is best characterized in the legume nitrogen fixing and mycorrhizal symbioses. More recently lipo-chitooligosaccharide (LCO) and thuricin 17, two microbe-to-plant signals, have been shown to regulate stress responses in a wide range of plant species. While thuricin 17 production is constitutive, LCO signals are only produced in response to a signal from the plant. We discuss how some signal compounds will only be discovered when root-associated microbes are exposed to appropriate plant-to-microbe signals (positive regulation), and this might only happen under specific conditions, such as abiotic stress, while others may only be produced in the absence of a particular plant-to-microbe signal molecule (negative regulation). Some phytomicrobiome members only elicit effects in a specific crop species (specialists), while other phytomicrobiome members elicit effects in a wide range of crop species (generalists). We propose that some specialists could exhibit generalist activity when exposed to signals from the correct plant species. The use of microbe-to-plant signals can enhance crop stress tolerance and could result in more climate change resilient agricultural systems.

## Introduction

Plants in nature are always in relationships (Raina et al., 2018) with a microbial community (the phytomicrobiome); some members of the soil microbial community assist plant growth and development (Prithiviraj et al., 2003; Smith et al., 2015a, b). The phytomicrobiome plus the plant constitute the holobiont—the holobiont is the entity that evolution acts upon, and that produces crop yield (Smith et al., 2017; Cordovez et al., 2019). When adaptation to environmental stressors is needed, the plant: (1) alters its own gene expression and resulting physiology, and also (2) adjusts the diversity, composition, and activity of its phytomicrobiome (Smith et al., 2015b; Gopal and Gupta, 2016). The latter allows for very short-term adjustments, including evolution of the phytomicrobiome; the plant genome evolves much more slowly (Mueller and Sachs, 2015). The genome of the phytomicrobiome (much larger than the plant genome) plus the plant genome comprises the hologenome or the pan-genome (the host plus the microbial metagenome) (Berendsen et al., 2012; Guerrero et al., 2013; Turner et al., 2013; Bordenstein and Theis, 2015).

It seems that evolution of more complex eukaryotic cells (Phylum Lokiarchaeota—Turner et al., 2013; Spang et al., 2015) from simpler prokaryotes, allowed development of the holobiont (Embley and Martin, 2006; Douglas, 2014; Koonin and Yutin, 2014; Graham et al., 2018). Beneficial relationships between terrestrial plants and microbes have existed since plants moved into the terrestrial environment, almost half a billion years ago (Knack et al., 2015). For about a billion years prior to this, algae had relationships with compatible microbial species, sometimes leading to new organisms. For example, *Ascophyllum nodosum* appears to be a fusion of a macroalga and a fungus (Deckert and Garbary, 2005).

The phytomicrobiome is tissue-specific and relationships vary in intimacy all the way to complete incorporation/fusion, as is the case with mitochondria and chloroplasts (Backer et al., 2018). The most abundant and diverse element of the phytomicrobiome is the rhizomicrobiome where microbes live around or within the root tissues, often in the spaces between cells of the cortex (the root is the niche space of these microbes), and use root exudates as a source of energy/reduced carbon (Schlaeppi and Bulgarelli, 2015). Rhizomicrobiome members can stimulate root growth and so improve plant water and nutrient uptake.

## Signal Exchange Between Plants and Microbes

The activity, diversity, and composition of the phytomicrobiome are often regulated by signal exchange between plants and microbes. This is best understood for the legume-rhizobia nitrogen fixation symbiosis; an isoflavonoid signal released from the plant is recognized by appropriate rhizobia that move up the concentration gradient toward the plant root. The isoflavonoid also triggers expression of nodulation and nitrogen fixation-related genes within appropriate rhizobia, some of which cause production of lipo-chitooligosaccharide (LCO) signals back to the plant (Lian et al., 2002). Detection of LCOs by the plant leads to nodule formation (Buhian and Bensmihen, 2018) and nitrogen fixation, once rhizobia have entered the nodule (Smith et al., 2015a, b; Bender et al., 2016). In some legumes, exposure to appropriate LCOs, in the absence of rhizobia, is sufficient to cause nodule formation; however, the nodules do not fix nitrogen. In another example, mycorrhizal fungi establish relationships with a wide range of plants (MacLean et al., 2017), mainly to facilitate uptake of soil phosphorus and, in some cases, contributing to the parasitism of other plants (e.g., some epiphytic orchids) (Latef et al., 2016). The plant produces strigolactones as a signal to the appropriate fungus and the fungus produces LCOs or similar compounds as return signals (MacLean et al., 2017). *Parasponia*, the only non-legume fixing nitrogen in symbiosis with rhizobia uses signals similar to the legume symbiosis (Behm et al., 2020) and the signals have been determined to be involved in establishment of the *Frankia* symbioses, although the exact identities are still unknown (Cissoko et al., 2018).

Research has demonstrated that molecular signaling between plants and members of the phytomicrobiome is involved in a large range of plant–microbe interactions. For example, our laboratory has shown that LCOs and thuricin 17 (a microbe-to-plant signal produced by *Bacillus thuringensis* NEB17) regulate plant growth and related activities, including abiotic stress responses (Smith et al., 2015a, b, 2017). Application of these signals cause expanded leaf area and increased photosynthetic rates (Almaraz et al., 2007; Khan et al., 2008); initial responses to these signals alter plant hormone profiles (Prudent et al., 2016). While specific LCO-crop species pairs exist in the context of legume nitrogen-fixing symbioses, there is evidence that the ability of LCOs to enhance plant stress tolerance is non-specific to crop species (Prudent et al., 2015; Smith et al., 2015a, b). This suggests that the role of LCOs in altering plant stress resilience is an older function than the signaling role in nitrogen fixation. We should anticipate this kind of two-way signal exchange in a reasonable proportion of beneficial plant–microbe relationships.

**FIGURE 1:**
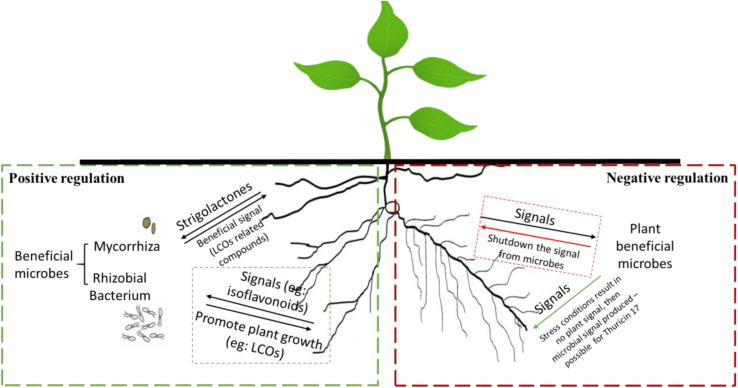
Examples of positive (left side) and negative (right side) regulation of microbe-to-plant signal production by plant-produced signals. **Positive regulation** (left): the plant root secretes a plant-to-microbe signal compound that activates microbe-to-plant signal compound production. **Negative regulation** (right): the plant constitutively produces a plant-to-microbe signal compound that inhibits production of a microbe-to-plant signal compound. When expression of the plant-to-microbe signal compound is downregulated, production of the microbe-to-plant signal compound occurs.

When a microbe-to-plant signal is required, perhaps due to abiotic/biotic stress conditions, the plant may produce a signal that triggers the release of a return signal by a microbe—this is **positive control** (Figure 1) (MacLean et al., 2017; Buhian and Bensmihen, 2018). Many microbes do not release microbe-to-plant signal molecules in culture, in the absence of the plant. However, addition of root exudates, which contain compounds that serve as plant-to-microbe signals, may induce the production of microbe-to-plant signal compounds. **Negative control** would occur when a plant-to-microbe signal compound inhibits microbial signal production; in the absence of the plant-to-microbe signal compound, the microbe produces a microbe-to-plant signal. This behavior might explain why *Bacillus thuringiensis* NEB17 produces large amounts of thuricin 17 when in culture (Subramanian and Smith, 2015), which is metabolically expensive.

**FIGURE 2:**
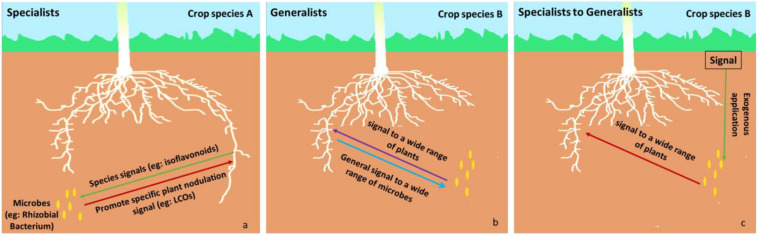
Examples of **specialist** (left side) and **generalist** (middle)**. Specialist (a):** a specific signal (red arrow, e.g., LCOs) is only expressed in the presence of a specific plant-to-microbe signal compound (green arrow, e.g., isoflavonoids) produced by specific *crop species A* (e.g., a specific legume). **Generalist (b):** a general signal (purple arrow) is expressed in the presence of a general plant-to-microbe signal compound (blue arrow) produced by a wider range of plants, such as *crop species B.* In some cases, **exogenous application (Specialist to Generalist c)** of a specific signal (green arrow) could result in the production of a microbe-to-plant signal by a microbe that usually functions as a specialist—the microbe-to-plant signal can be recognized by a wide range of plant species and the microbe is converted from a specialist into a generalist one (e.g., if the plant-to-microbe signal from *crop species* A is applied to a microbe in the presence of *crop species B*). For example, **exogenous application** of a specific plant-to-microbe signal (e.g., genistein, an isoflavonoid from soybean, in a legume nitrogen-fixing symbiosis) results in the production of the microbe-to-plant signal (e.g., LCO) in the rhizosphere of a wide range of plants, where the microbe-to-plant signal has an alternative function (e.g., regulation of plant stress responses).

Members of the phytomicrobiome can be categorized as generalists or specialists depending on the range of plant species, they elicit effects from: **specialists** affect a narrow range of plant species whereas **generalists** affect a wide range of plant species (Figure 2). For example, rhizobia produce LCO signals that are extremely plant species-specific during establishment of the nitrogen-fixation symbiosis (Poustini et al., 2007; Clúa et al., 2018). This constitutes a specialist effect. In contrast, when LCOs promote stress resilience across a wide range of plant species, this constitutes a generalist effect (Smith et al., 2015a, b). Specialists may only exert their effects in the presence of a plant-to-microbe signal compound which is, perhaps, only excreted by plant roots under specific conditions (e.g., nodulation, abiotic or biotic stress). This induces production and release of the microbe-to-plant signal compound into the rhizosphere. An example of a specialist microbe-to-plant signal is lumichrome which is produced by the degradation of riboflavin by specific microbes, such as *Pseudomonas* (Yanagita and Foster, 1956) and *Sinorhizobium meliloti* (Phillips et al., 1999), and promotes the growth of certain crops (Rovira and Harris, 1961; Sierra et al., 1999; Dakora, 2015). It may also be possible for a microbe or its signal to be switched from specialist to generalist. For example, LCO is usually only produced by rhizobia in response to a microbe-to-plant signal molecule. However, when exogenous genistein is added to the rhizobial culture, the bacteria produce LCO even in the absence of living rhizobia. This LCO can then stimulate plant growth in a range of crop species (Smith et al., 2015a, b).

## Evolved Benefits of Plant–Microbe Interactions

When a microbe provides a strong benefit to a plant, the microbe and the plant have probably coevolved for a long time (Wallenstein, 2017) and the microbe may provide multiple benefits to the plant. For instance, microbes that help with abiotic or biotic stress resistance (through priming of stress response pathways, competition or antagonism against plant pathogens) may also assist in plant nutrient acquisition (N fixation, production of siderophores, P solubilization) (Fan et al., 2018). Members of the phytomicrobiome may coordinate activities to increase plant root exudation which benefits the whole microbial community; simultaneously, members of the phytomicrobiome compete for resources, including the niche space provided by plant roots. The microbes may produce compounds that inhibit microbial activity, such as antibiotics. For instance, bacteriocins are proteins synthesized by bacteria; bacteriocins kill closely-related strains, thereby minimizing competition from strains with the greatest metabolic similarity (Gray et al., 2006a, b). Thuricin 17 is both a bacteriocin and a microbe-to-plant signal compound that improves plant stress resilience and is thus a dual-function protein (Subramanian and Smith, 2015). This imposes constraints on evolution—a process that is always pragmatic, random, relentless, and ruthless—to maintain thuricin 17 production due to its multiple biological activities that benefit the microbe. The genome of *B. thuringiensis* strain NEB17 contains three tandem repeats of the gene that produces thuricin 17 and the copies have evolved differences. However, all the nucleotide differences are found in the third codon position and code for amino acid redundancies. So, while the nucleotide sequences vary among the gene copies, the amino acid sequence of the proteins does not (Gray et al., 2006a, b), illustrating the evolutionary conservatism resulting from the dual function nature of the protein encoded by the gene.

Plants have also evolved to recognize and respond to signals exchanged between members of the phytomicrobiome. For example, lactones, which are used as inter-microbial signals in quorum sensing, are monitored and responded to by plants (Ortiz-Castro and López-Bucio, 2019), possibly because biofilms, potentially produced as a result of quorum sensing, can provide benefits for plant growth (Hartmann and Rothballer, 2017; Ricci et al., 2019). In addition, plants and phytomicrobiome members communicate through many volatile organic compounds (VOCs; Lee et al., 2016; Kashyap et al., 2017; Liu and Brettell, 2019). For example, an immobile bacterium that lives in the phyllosphere produces a volatile signal to call over a mobile bacterium, to carry the immobile one along (Hagai et al., 2014).

Some members of the phytomicrobiome deter microorganisms that damage plants, or compete for resources (Droby et al., 2016; Ab Rahman et al., 2018). With increasing concern around environmental impacts of chemical pesticides (Bender et al., 2016), members of the phytomicrobiome that produce compounds bacteriostatic or bactericidal to plant-detrimental organisms are of commercial interest (Ab Rahman et al., 2018; Anderson and Kim, 2018). The Bt toxin, originally from a *B. thuringiensis* strain, was genetically engineered into a wide variety of crops because of its insect control activity. Work in our laboratory has shown that the *B. thuringiensis* strain producing thuricin 17 also produces the very effective insecticide beta-exotoxin. In addition, we have recently isolated a pair of compounds, produced by a plant growth-promoting rhizobacteria (PGPR), that are effective against a tomato pathogen (Takishita et al., 2018). These are examples of compounds produced by the phytomicrobiome that can be commercialized to improve crop productivity. In addition, biocontrol organisms can also produce compounds that trigger plant immune responses which represents an alternative mechanism for pathogen control in crops (Ab Rahman et al., 2018; Takishita et al., 2018).

Because evolution never sleeps, signal exchange systems between plants and beneficial microbes have been exploited by parasitic organisms. For example, spores of the pathogen *Phytophthora* can detect isoflavonoid signals from soybean roots and swim up the concentration gradient to find the roots (Hua et al., 2015; Zhang et al., 2019). In addition, seeds of the parasitic plant *Striga* germinate when they detect the plant-to-microbe strigolactone signals, indicating proximity to host-plant roots (Yoneyama et al., 2019). This is a serious problem for crop production in some areas of the world.

## The Phytomicrobiome and Plant Stress

Effects of PGPR on plants can be inconsistent (Nelson, 2004). One possible explanation is that plant growth responses to many PGPR interact with plant stress (Smith et al., 2015a, b; Ilangumaran and Smith, 2017; Vimal et al., 2017; Backer et al., 2018). It is possible that in some cases when PGPR were reported to have effects on plant growth, the plants were growing under stressful conditions and PGPR improved stress resilience. This could have occurred as a result of seemingly benign factors such as, the timing of watering during experiments (leading to intermittent drought stress) or spikes in greenhouse temperatures (leading to acute heat stress). Plant phosphate stress responses may also shape the root microbiome in turn affecting plant immunity (Castrillo et al., 2017). Likewise, salicylic acid, involved in plant stress responses, is also essential for endophytic root microbiome assembly (Lebeis et al., 2015).

Under stress, a plant can: (1) become resilient or (2) become dormant (close stomata, senesce tissues—e.g., leaves under severe drought) (Considine and Considine, 2016). The second option is often associated with elevated levels of plant hormones such as abscisic acid and ethylene. From the perspective of the bacteria, it is desirable that the plant remains resilient, the first option, photosynthesizing and continuing to produce root exudates which serve as a carbon source for PGPR (Backer et al., 2018). Thus, there can be **dynamic tension** between those dependent on plant productivity (phytomicrobiome members and agriculturalists) and the plant, when it faces stress. An example of this would be the regulation of ethylene production from 1-aminocyclopropane-1-carboxylic (ACC) acid by members of the phytomicrobiome. Plant-associated microbes produce ACC deaminase, which prevents the final production of ethylene from ACC (Glick, 2014). This maintains low ethylene levels in the plant, and the plant is less likely to become dormant (Backer et al., 2018). Thus, the phytomicrobiome diverts plant activity to best suit microbial requirements for growth by improving plant nutrient availability and eliciting plant stress responses.

Application of LCO, thuricin 17, jasmonic acid, and VOCs to plants growing under stressful conditions has been reported to improve plant resilience to stress (Subramanian and Smith, 2015; Prudent et al., 2016). When LCOs were sprayed onto leaves of stressed soybean plants (growing at 15°C), stress response genes were the largest class of known-function genes with altered expression levels (Wang et al., 2012). This suggests that the plant switches from one set of stress response genes to another, perhaps, from genes related to dormancy to those related to stress resilience. Subsequent research revealed that treatment with LCO and thuricin 17 (Subramanian and Smith, 2015) increased levels of stress-related and energy metabolism proteins (Subramanian et al., 2016a, b). However, this strategy can go too far as the application of higher concentrations of LCO and thuricin 17 to very stressed plants can result in plant mortality (unpublished data). Other compounds such as jasmonic acid, a hormone involved in plant stress responses, also trigger LCO production by rhizobia (Mabood and Smith, 2005; Mabood et al., 2006b; Smith et al., 2015a). In addition, there are reports of VOCs enhancing plant stress tolerance (Kashyap et al., 2017).

## Contribution of the Phytomicrobiome to Global Food Security

The phytomicrobiome and the signal compounds exchanged between plants and microbes play a key role in determining crop yields, particularly in the presence of challenges such as a/biotic stresses (Mueller and Sachs, 2015; Wallenstein, 2017), including those associated with climate change (drought, high temperature, flooding, salinity) (Almaraz et al., 2008; Smith and Zhou, 2014; Kashyap et al., 2017; Vimal et al., 2017; Backer et al., 2018). At a time when we are concerned about the environmental impacts of pesticides (Busby et al., 2017) and extensive fertilizer application, PGPR and microbe-to-plant signal molecules offer alternative strategies for increasing, or at least maintaining, crop yields with reduced pesticide and fertilizer inputs while developing more climate change-resilient agricultural systems. There is enormous potential in our ability to manipulate the phytomicrobiome and its signals, as our understanding of this very complex system grows (Mabood et al., 2006a; Schlaeppi and Bulgarelli, 2015; Smith et al., 2015b; Gopal and Gupta, 2016; Głodowska et al., 2017; Lamont et al., 2017; Wallenstein, 2017). In addition, LCO and thuricin 17 are effective at very low concentrations (LCOs: 10^–6^–10^–8^ M, thuricin 17: 10^–9^–10^–11^ M; Smith et al., 2015a, b; Subramanian and Smith, 2015) and are inexpensive to produce. The LCO technology is already being applied to tens of millions of hectares of agricultural land each year. The phytomicrobiome can contribute to the effort for global food security.

## The Future of Plant–Microbe Interaction Research

To identify new beneficial strains from the phytomicrobiome, or microbe-to-plant signal compounds, one must have clear objectives, for example: (1) to reduce the impact of stress on crop yields, (2) to reduce fertilizer application, and/or (3) to reduce disease impacts. Rapid and effective screening methods to identify promising microbes and/or microbe-to-plant signals are required (Mueller and Sachs, 2015; Backer et al., 2018). Generalist strains could be isolated from a wide range of plant species; our laboratory has isolated agriculturally useful PGPR from undomesticated plant species. While the phytomicrobiome of domesticated plants is under-investigated, that of undomesticated plants remains very unexplored. Furthermore, exciting discoveries under laboratory conditions may not always prove effective under field conditions since we do not understand all of the nuances of this highly complex and regulated system (Backer et al., 2018). The various natural environments contain a large indigenous community of microbes, experience a wide range of environmental conditions, and vary in soil properties from site to site (Sessitsch et al., 2019) so that a wide range of potential plant-beneficial microbes probably occur in non-agricultural settings.

New methods will have profound effects on research related to phytomicrobiome signaling and plant growth. **Phenotyping** allows determination of subtle but key effects on plants/holobionts, providing the capacity to determine features like space occupancy, in relation to plant light interception (Walter et al., 2015; Lopes et al., 2018). Newer **CT scanning** applications allow for determination of space occupancy and fractal dimensions of undisturbed roots in soil (Costa et al., 2003; Dutilleul et al., 2005, 2008; Lontoc-Roy et al., 2005; Han et al., 2008; Subramanian and Smith, 2015).

One should not fall into the trap of assuming that the effect(s) of a novel growth-stimulating microbe must result from previously established mechanisms (Backer et al., 2018). There will be novel signals with new and surprising new modes of action (Hagai et al., 2014). At this time, we have narrow understanding of how a tiny fraction of plant–microbe interactions occur and coordinate the activity of the holobiont. There is a breathtaking amount to learn.

## Author Contributions

DS gathered literature and prepared the manuscript. DL contributed to the initial writing and structuring of the manuscript. RB and SS provided feedback and oversaw progression of the manuscript. All authors gave final approval for publication and agreed to be held accountable for the work contained therein.

## Conflict of Interest

The authors declare that the research was conducted in the absence of any commercial or financial relationships that could be construed as a potential conflict of interest.
